# The Use of ^99m^Tc-Mononuclear Leukocyte Scintigraphy for Necrotizing External Otitis Diagnosis

**DOI:** 10.3390/diagnostics13030570

**Published:** 2023-02-03

**Authors:** Sergio Augusto Lopes de Souza, Roberta Silveira Santos Laurindo, Gabriel Gutfilen-Schlesinger, Felippe Felix, José Luiz de Medeiros Amarante Junior, Bianca Gutfilen

**Affiliations:** 1 Serviço de Otorrinolaringologia, Hospital Universitário Clementino Fraga Filho, Universidade Federal do Rio de Janeiro, Rio de Janeiro 21941-913, Brazil; 2 Departamento de Radiologia, Faculdade de Medicina, Universidade Federal do Rio de Janeiro, Rio de Janeiro 21941-913, Brazil

**Keywords:** external otitis, leukocyte scintigraphy, differential diagnosis

## Abstract

Background: Necrotizing external otitis (NEO) is a severe infectious disease in the external acoustic meatus (EAM) and mastoid that may extend to the cranial base. Due to the lack of a gold standard examination technique, the diagnosis is often difficult and delayed. This study aimed to evaluate the sensitivity and specificity of ^99m^Tc-mononuclear leukocyte scintigraphy associated with ^99m^Tc-phytate in suspected NEO compared to ^99m^Tc-MDP and ^67^Ga-citrate. Methods: A prospective study (32 patients) was conducted between 2011 and 2016. Results: At the end, twenty-four patients remained for the study conduction; nineteen had confirmed NEO diagnosis, one had sarcoma, one had EAM cholesteatoma, one had diffuse simple external otitis, and two had an inconclusive diagnosis. ^99m^Tc-mononuclear leukocyte scintigraphy plus ^99m^Tc-phytate was as sensitive as ^99m^Tc-MDP bone scintigraphy (19/19X9/19), and more sensitive than ^67^Ga scintigraphy (19/19 x 17/19). Regarding specificity, it was superior to bone scintigraphy, 100% × 40% (5/5 × 2/5), and ^67^Ga scintigraphy, 100% × 20% (5/5 × 1/5). After the infection resolution, all NEO patients had their leukocyte scintigraphy negativized. To the best of our knowledge, this is the first study that evaluates this technique in patients with suspected NEO. Conclusions: ^99m^Tc-mononuclear leukocyte was revealed to be the best option for NEO because of its specificity.

## 1. Introduction

Necrotizing (malignant) external otitis (NEO) is a severe infection in the external auditory meatus (EAM) and mastoid with a potential expansion to the base of the skull [1]. It is mainly affecting immunodeficient and diabetic patients [2,3,4].

The clinical course of NEO is as unpredictable as its prognosis. Due to the rarity of this disease, it poses a clinical challenge. Evidence-based knowledge is derived from a small case series or historical cohorts in which diagnosis is based mostly on physical examination and response to treatment [2,3,4]. Previous attempts were made to establish diagnostic criteria, prognostic factors, and treatment protocols without, reaching a consensus [5,6]. Nowadays, the diagnostic protocol combines clinical, laboratory, radiological, and, nuclear medicine findings. Differential diagnosis includes chronic otitis media and neoplasia. Hence, distinguishing NEO from other diseases is not a simple task [2,3,4,7,8].

Nuclear medicine techniques are essential for diagnostic and treatment efficiency [7,8]. Bone scintigraphy with ^99m^Tc-MDP is not very specific, but its sensitivity can detect any bone alteration. Meanwhile, gallium-67 scintigraphy can measure patient treatment response, whereas it cannot be used for osteomyelitis diagnosis [9].

^99m^Tc-leukocytes scintigraphy was developed 30 years ago and it is helpful in infectious foci, osteomyelitis, fevers of unknown origin, and differential diagnoses between loose or infected prostheses [10,11]. A useful tool for the latter is associating labeled leukocytes with phytate (a colloid mainly used for bone marrow integrity evaluation). This combination provides heightened specificity and sensibility [12].

Both reflect the radiotracer accumulation in the endothelial reticulum cells in the bone marrow. However, whenever infected tissue is present, its distribution profile changes. This inversion phenomenon culminates in a resulting incongruent image, which allows differentiating inflammation from infection [12].

A question remains in the literature: what is the best test for diagnosis and monitoring treatment response in NEO? Concerns are also raised regarding the accuracy and cost-effectiveness of the current imaging modalities presented.

Thus, this study aimed to evaluate the sensitivity and specificity of ^99m^Tc-mononuclear leukocytes scintigraphy (^99m^Tc-MLS), enhanced with ^99m^Tc-phytate scintigraphy in comparison with ^99m^Tc-MDP and ^67^Ga scintigraphy.

## 2. Materials and Methods

A prospective study was conducted between 2011 and 2016 at the Hospital Universitário Clementino Fraga Filho (HUCFF/UFRJ), Rio de Janeiro, Brazil, with patients with NEO suspicion. These cases underwent a protocol created by the Services of Otorhinolaryngology, Hospital Infection Control, and Nuclear Medicine of the institution.

Inclusion criteria: Patients with NEO clinical suspicion were included in this study when one of the following factors was found:

(1) deep-seated otalgia, relentless or aural fullness sensation persisting for more than one week; (2) physical examination according to external otitis (edema, hyperemia in EAC with or without otorrhea); and (3) signs and symptoms persisting even after one week of antibiotic treatment for acute external otitis.

Patients who did not perform ^99m^Tc-MLS at the beginning of the protocol or abandoned the treatment were excluded from this work.

**Figure 1:**
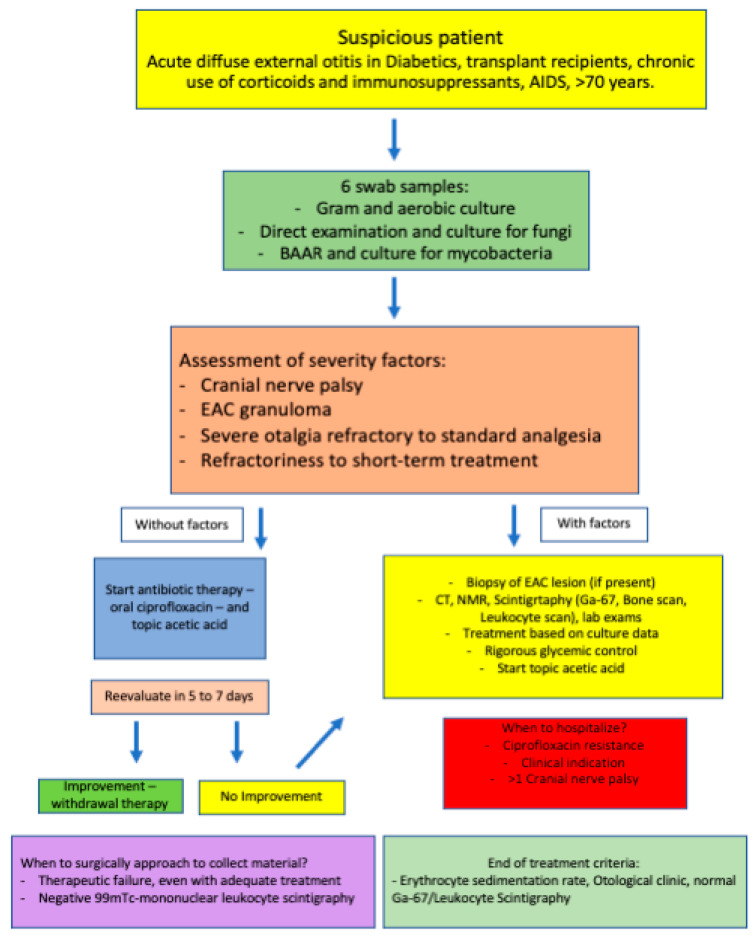
Suspected NEO patient management.

Suspected NEO patient management: After raising suspicion, six single-swab samplings of the EAM collection were made for microbiological purposes, including gram, aerobic, direct exams, mycological cultures, culture, and alcohol acid-fast bacilli (AAFB) for mycobacterial cultures (Figure 1).

The following severity signs were evaluated: cranial nerve palsy, EAM granuloma, severe refractory otalgia to standard analgesia, and refractoriness to short-term conventional treatment with antibiotics for external otitis.

When present, at least one of the listed above signs mentioned protocol was started (NEO differential diagnoses and antibiotics treatment). Otherwise, ciprofloxacin was given at 750 mg twice daily (12/12 h) and topic 2% acetic acid in an aqueous solution thrice daily (8/8 h). Afterward, the patient was re-evaluated in 5–7 days. Treatment was interrupted if improvement was noticed and the patient was discharged with an acute external otitis diagnosis. If one of the signs or symptoms was still present, the NEO investigation protocol was continued.

### 2.1. Investigatory Protocol for the NEO Diagnostic

(1) Lesion biopsy of the EAM (when present); (2) lab work: blood counts, fasting glycemia, glycated hemoglobin, urea, creatinine, ESR, and CRP; (3) mastoids computed tomography; (4) mastoids magnetic resonance imaging; (5) ^99m^Tc-MDP (20mCi) scintigraphy 3 h after radiotracer injection planar and tomographic (SPECT) images were taken. (6) One week after the bone scan, a ^67^Ga (10mCi) scintigraphy was acquired (planar and SPECT) 24 and 48 h after radiotracer injection. (7) One week after ^67^Ga scintigraphy, a ^99m^Tc-mononuclear leukocytes (15mCi) scintigraphy was acquired as follows: 1, 3, and 24 h after labeled cell injection planar and tomographic (SPECT) images were taken. A total of 24 h later, ^99m^Tc-phytate (5mCi) scintigraphy was performed for bone marrow integrity evaluation. Planar scans had a 5 min acquisition time and SPECT scans had an 8 min acquisition time. (8) Acquired data were then compared for proper patient management. The whole image protocol was always in the following order: bone, gallium, and leukocyte scintigraphy. The whole diagnostic procedure lasted around 15 to 20 days. The main criterion for positivity was comparative visual uptake.

### 2.2. Treatment and Management of the Suspected NEO

The first therapeutic approach was ciprofloxacin 750 mg given twice daily (12/12 h). Culture biopsies from the EAM were collected before starting the antibiotics therapy. If the culture findings were compatible with the ciprofloxacin-resistant microorganism, it was shifted to another one according to the antibiogram. If the patient was not responsive to treatment even with non-resistant microorganism lab results, intravenous (IV) cefepime was used instead of ciprofloxacin. Associated with systemic antibiotics, topic 2% acetic acid in an aqueous solution (3 drops thrice a day) and meticulous glycemic control (in case of DM) were conducted. If the arriving patient had already begun treatment, EAM biopsy swabs were taken, and switching therapeutics were concerned with the other centers involved.

Patient hospitalization was considered if one of the following factors were present: IV antibiotics were needed, uncontrolled DM, dysphagia, more than one cranial nerve palsy, or inability to return for further investigation. The initial criteria for the biopsy procedure were EAM lesion presence. Biopsy was made at the outpatient healthcare, with local anesthetics. Therefore, patients were without visual EAM lesions, although collection swab samples were made for culture and antibiogram analysis. Biopsy was conducted for all patients with negative 99mTc-MLS and/or therapeutic failures, even with proper antibiotics therapy. Those patients underwent a tympanomastoidectomy under systemic anesthesia for soft and hard tissue collection. The retrieved material was sent for histopathological and culture analysis.

### 2.3. Healthcare Follow-Up and Healing Criteria

In outpatient cases, the returning frequency for clinical evaluation and the aural toilet was weekly until the erythrocyte sedimentation rate (ESR), clinic otological exam, and both scintigraphies (^99m^Tc-MLS and ^67^Ga) were normal. ^99m^Tc-MLS and ^67^Ga scintigraphies were acquired every four weeks, while ESR and clinical exams were checked weekly. Bone ^99m^Tc-MDP scintigraphy was not used for treatment responsiveness due to its low specificity, as is established in the literature.

### 2.4. The 99mTc-Mononuclear Leukocytes Scintigraphy Technique

The methodology used in this work to label mononuclear leukocytes with ^99m^Tc was the one described by Gutfilen et al. (11). Briefly, 20 mL of blood is collected in sterilized tubes treated with heparin. For mononuclear cell sorting, Ficoll-Hypaque is used. Then, the remaining cells are washed with saline (0.9% NaCl) and centrifuged. Pellet is resuspended in saline and is microcentrifuged for platelet removal. The final cell concentration is 10^7^/mL. Cells are then incubated in stannous chloride (SnCl_2_ 2H_2_O, Merck, Brazil) for 10 min at room temperature. Afterward, ^99m^Tc (370 MBq) is added to the solution and incubated for another 10 min. The resulting pellet is resuspended in saline and injected into the respective patient. Scintigraphy is then acquired immediately, 30 min, 3, and 24 h after cell injection.

^99m^Tc-MLS consisted of a whole body (as quality control), anterior, posterior, and lateral projections 1, 3, and 24 h after the administration of the labeled cells. SPECT scans were acquired as complementary images. Planar scans had a 5 min acquisition time and a 256 × 256 matrix, the whole body had a 15 min acquisition time and a 256 × 1024 matrix, and SPECT had a contoured orbit, a 128 × 128 matrix, 6-degree intervals, and a 15 sec/stop with a reconstruction made using OSEM, and a Butterworth filter with a cut-off 0.4 and power 7.

#### Resulting ^99m^Tc-MLS Analysis with Complementary ^99m^Tc-Phytate Scintigraphy

In the case of positive ^99m^Tc-MLS, complementary ^99m^Tc-phytate scintigraphy was conducted. Both procedures reflect radiotracer accumulation in bone marrow reticuloendothelial cells. However, their distribution pattern differs when infected tissue is present. These inverted effects generate incongruent images concluding an infection diagnosis. Thus, when both scintigraphy results are positive, it is possible to rule out an infection scenario even though there is still inflammation at the examined site.

## 3. Results

This study started with thirty-two patients, but during its course, eight were excluded (seven for not completing the ^99m^Tc-MLS and one for abandoning the treatment), resulting in a total of twenty-four cases for analysis. The average patient (nineteen males, four females) age was 54.3 years old, ranging from 14 to 73. One patient presented bilateral disease. Comorbidities found within the group included twenty-two with DM, one with rheumatoid arthritis, chronic kidney disease, and one with lymphoma. From the twenty-four initial NEO suspicion diagnoses, nineteen were confirmed afterward; one had sarcoma, one had cholesteatoma of EAM, one had otitis externa, and two had inconclusive diagnostics. One of the latter died due to a pulmonary embolism.

### 3.1. Laboratory Work

*Pseudomonas aeruginosa*, *Klebsiella pneumoniae, Staphylococcus* coagulase-negative, and *Aspergillus* were the etiological agents in a 9:1:1:1 ratio, respectively, in the NEO confirmed cases, while seven had negative culture results.

### 3.2. Scintigraphy

**Figure 2:**
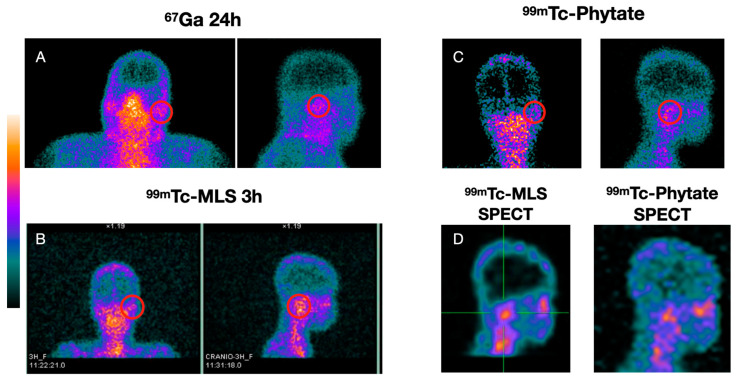
Patient 6. A male, 21 year-old patient with acute myeloid leukemia in remission with NEO suspicion in his right ear. Returning lab results revealed *Pseudomonas aeruginosa* positive culture, although no improvement was found after antibiotics treatment. Bone ^99m^Tc-MDP and ^67^Ga scintigraphy (**A**) were positive at the right mastoid topography. Both findings are in agreement with the infectious–inflammatory process in the temporal bone hypothesis. High uptake in ^99m^Tc-MLS in right mastoid topography (posterior and lateral incidence) 1 and 3 h (**B**) after labeled cells injection and enhanced with ^99m^Tc-phytate showing uptake in the same topography (**C**). SPECT (**D**) from ^99m^Tc-MLS and ^99m^Tc-phytate revealing the agreement in the uptake putting aside the infectious process possibility. After 6 weeks of antibiotics, a neoplasm was considered, and a biopsy revealed a myeloid sarcoma.

**Figure 3:**
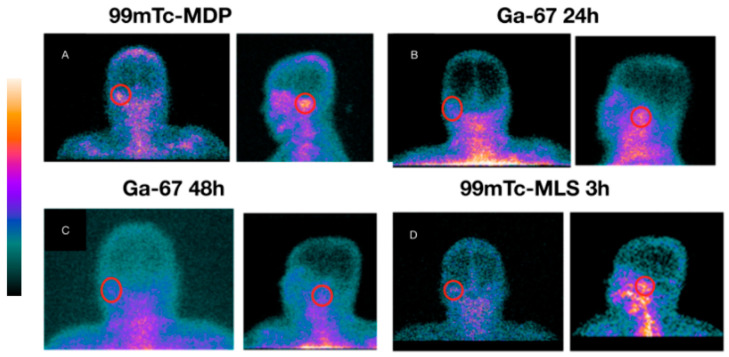
Patient 11. (**A**) Bone ^99m^Tc-MDP scintigraphy in left mastoid topography (posterior and lateral incidence). (**B**) ^67^Ga scintigraphy taken 24 h after radiotracer iv. injection showing higher uptake at the region of interest (ROI, red ring). (**C**) ^67^Ga scintigraphy taken 48 h after radiotracer iv. injection showing a long-lasting higher uptake at the ROI. (**D**) ^99m^Tc-MLS with a higher uptake 3 h after iv. injection of labeled cells. A skin biopsy showed a chronic nonspecific inflammatory process characterized by granulation tissue with recently formed blood capillaries, fibroblasts, and infiltrated leukocytes at the site.

**Figure 4:**
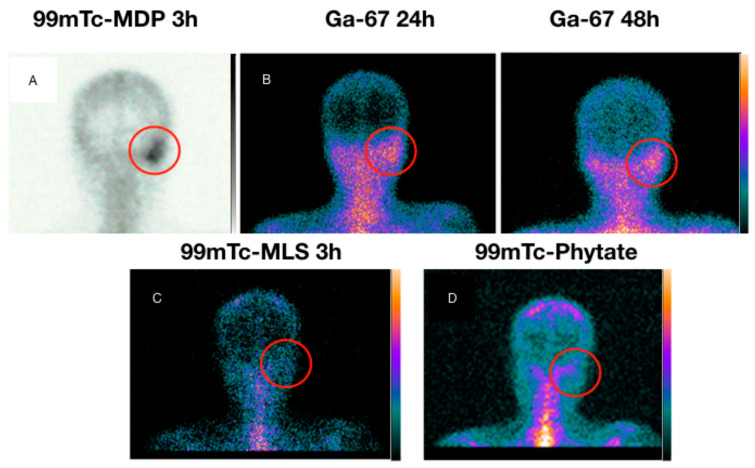
Patient 19. Male patient, 38 years old, DM insulin-dependent, with right otalgia and refractory otorrhoea at the beginning of treatment. Otoscopy revealed small erosion in the posterior EAM, which is associated with granulation tissue and pus. Culture analysis results revealed Proteus mirabilis ciprofloxacin sensitivity. Acquired images in anteroposterior incidence. (**A**) Bone ^99m^Tc-MDP scintigraphy in right mastoid topography (ROI, red ring). (**B**) ^67^Ga scintigraphy in 24 h and 48 h after iv radiotracer injection, which shows persistent uptake. (**C**) ^99m^Tc-MLS was inconsistent with the infection hypothesis. (**D**) ^99m^Tc-phytate scintigraphy showing uptake at ROI. Histopathology revealed a cholesteatoma in EAM.

Table 1 shows all image results with the final diagnosis. The sensitivity, specificity, and accuracy for ^67^Ga scintigraphy were 89% (17/19), 20% (1/5), and 75% (18/24), respectively. Bone ^99m^Tc-MDP scintigraphy sensitivity, specificity, and accuracy found were 100% (19/19), 40% (2/5), and 87,5% (21/24), respectively. ^99m^Tc-MLS sensitivity, specificity, and accuracy were 100% (19/19), 80% (4/5), and 95,8% (23/24), respectively. When enhanced, the ^99m^Tc-MLS with 99mTc-phytate resulting sensitivity, specificity, and accuracy were 100% (19/19), 100% (5/5), and 100% (24/24), respectively. Figure 2 shows a patient with acute myeloid leukemia in remission with NEO suspicion in his right ear. Laboratory results revealed *Pseudomonas aeruginosa* positive culture, although no improvement was found after antibiotics treatment. Figure 3 shows a DM insulin-dependent female patient with left otalgia and refractory otorrhoea with NEO suspicion. Figure 4 shows a DM insulin-dependent patient with right otalgia and refractory otorrhoea at the beginning of treatment. Otoscopy revealed minor erosion in the posterior EAM, associated with granulation tissue and pus. Culture analysis results showed *Proteus mirabilis* ciprofloxacin sensitivity. All patients with definitive NEO diagnosis had scintigraphy with negative results after infection clearance.

## 4. Discussion

NEO is a challenging condition to conclude the diagnosis. This is an important limitation regarding all conducted studies on this illness, including ours. If the studied entity is not well-defined, any discussion about its management is jeopardized. Many cases need to be better characterized based on NEO’s definition. The lack of a gold-standard diagnostic and patient management tool also compromises early treatment outset [13]. Although the late diagnosis is not a life-threatening factor, it heightens comorbidities and patient discomfort [13,14,15,16].

A rare disease makes it challenging to accumulate many patients to achieve a higher statistical trust/potency value. Despite that, the present work achieved a statistically relevant group for early ^99m^Tc-MLS technique evaluation as a diagnostic tool. We also managed to compare it to other methods used for this purpose.

Computed tomography (CT) is taken as a quick and easy, commonly used technique for mastoid region assessment [7,17,18]. Characteristic NEO initial findings during clinical examination are injury oozing edema near the Santorini fissure. Unfortunately, in those early stages of the disease, CT is nonspecific because it only highlights soft parts edema and adipose tissue thinning^7^. It is noteworthy that in the early stages of osteomyelitis, CT does not always show bone degradation at the cranial base. This phenomenon is a relatively late-set feature^7^. Hence, a clear CT in early disease stages does not exclude a NEO diagnosis. Meanwhile, bone ^99m^Tc-MDP scintigraphy highlights osteitis even before bone erosion could be seen on CT [10,19,20,21].

An MRI is a better choice for treatment response follow-up studies due to its soft tissue and bone marrow resolution capabilities instead of using CT for the same purposes [22,23]. A conventional MRI of the Achilles’ heel is has limited precision in distinguishing active infection from post-inflammatory fibrosis changes and granulation tissue, which can last longer than one year [3]. A recent study evaluated MR-weighted images in seven NEO patients with promising results, finding a relationship between the apparent diffusion coefficient from the inflammation signal resolution, clinical resolution, and infection biochemistry [24].

The most used nuclear medicine tools for NEO patients are ^99m^Tc-MDP and ^67^Ga scintigraphy [9]. Nowadays, some studies have evaluated FDG-PET for NEO assessment [7,25]. We did not find in the literature ^99m^Tc-HMPAO-leukocytes being used specifically in NEO cases, only for osteomyelitis [26].

Bone ^99m^Tc-MDP scintigraphy is a top-notch diagnostic tool with excellent sensitivity for osteomyelitis, as radiotracer accumulation occurs in areas of intense osteoblastic activity, which is also seen in neoplasias, post-surgery, and in external otitis [21]. It remains positive even after full recovery from the process, it cannot differentiate bone remodeling from active infection, and thus cannot be used as a follow-up or therapy response marker [18].

In our study, ^99m^Tc-MLS labeling and diagnostic techniques revealed to be as sensitive as ^99m^Tc-MDP scintigraphy, with higher specificity (80% vs. 40%) in NEO cases. When enhanced with 99mTc-phytate, this measurement rises to 100%, demonstrating how outstanding performance can be reached by combining the two labeling techniques for a better distinction of infection, bone inflammation, and neoplasia.

Macrophages and reticular endothelial cells absorb ^67^Ga, being found in inflammatory sites whether in bone, soft, or infectious tissues [27]. ^67^Ga scintigraphy does not last after infection clearance; hence, it can be used in radiology as a therapeutic response marker [9]. However, ^67^Ga scintigraphy has a higher cost, is more time-consuming, and has higher radiation emission if compared with bone or leukocyte scintigraphy [28,29]. Regarding osteomyelitis diagnosis, ^67^Ga scored 70% for sensitivity and 93% for specificity [2]. Here, we found similar results from the respective literature, excellent too in treatment control, with 89.4% sensitivity, and discording with the literature in the specificity aspect, we could only find 20% effectiveness in this matter for all the NEO analyzed cases. Meanwhile, ^99m^Tc-MLS reached 100% marks in both aspects.

Labeled leukocytes have been showing promising results in osteomyelitis evaluation studies. Phytate is also being used to aid in distinguishing infection from inflammatory processes and can also be handy providing complementary data for leukocyte scintigraphy.

In some of our cases, phytate played a major role in pointing out the right mastoid topography, being the only feedback to discard the infection hypothesis, later confirmed by the biopsy result of sarcoma and cholesteatoma, respectively.

^111^in-granulocyte-oxin and ^99m^Tc-HMPAO-leukocyte scintigraphy are well-established imaging techniques for infection and inflammatory bowel disease. Though, their findings in the literature suggest that mononuclear cells are better diagnostic tracers than neutrophils implemented in the ^99m^Tc-HMPAO technique. In chronic osteomyelitis, for instance, bacterial antigens activate lymphocytes and macrophages (previously monocytes), not involving neutrophil recruitment; hence, they are undetectable with the ^99m^Tc-HMPAO technique [10,11,26]. The leukocyte scintigraphy method used in this study demonstrates the advantage of using mononuclear immune cells (lymphocytes and monocytes), achieving higher accuracy for chronic infectious processes, such as NEO.

^99m^Tc-MLS is a simple technique with a key role in locating inflammation and infection. In osteomyelitis and infectious cases, in previously published data from our group [10,11], it achieved 69% and 100% in sensitivity and specificity, respectively, when compared to laboratory exams, hemoculture, histopathology, and radiology results (MRI and CT) [1]. It is still considered the gold standard for post-traumatic or operatory chronic osteomyelitis [30,31].

Among its advantages, we can point out that the blood amount needed is very little (20 mL), it is a faster cell labeling procedure (1–1.5 h) with no use of forbidden substances, and it is low cost and it provides the mononuclear fraction labelling [10]. It is being used for infectious foci, osteomyelitis, myocarditis, implantable devices infection, the differential diagnosis between kidney rejection transplant and acute tubular necrosis, the differential diagnosis between loose prosthesis and infection, and for patients with fever of unknown origin [11].

Our study shows that ^99m^Tc-MLS has relevance mainly in infection, inflammation, and neoplasia distinction. Its high specificity is a powerful tool for NEO diagnosis. In three cases presented within our study, it was the only method that could discard infection and corroborate with the histopathological report. In the two cases without a conclusive diagnosis, it did not support the infection hypothesis, thus discarding NEO and enabling the antibiotic therapy interruption without worsening patients’ clinical scenario.

Autologous leukocyte scintigraphy, labeled with ^111^In or ^99m^Tc, is still considered the gold standard for infection or inflammation, but the availability of radiolabeled compounds database is in constant and quick expansion. The results from the current work support this data, as we provided enough evidence that ^99m^Tc-MLS is an excellent diagnostic tool for NEO with higher specificity when compared with ^99m^Tc-MDP and ^67^Ga- scintigraphy, which are the most used labeling techniques nowadays, for this end.

Lately, the use of positron emitters has been evaluated, and according to the literature, they present enhanced resolution. This technique is based on the principle of coincidental detection, which considerably heightens spatial resolution in comparison with conventional (gamma) imaging. However, the main positron emitter used in infectious diseases, ^18^F-FDG (2-fluoro-2-deoxyglucose), is responsible for detecting metabolic changes in case of infection. Nevertheless, it is also characterized by a metabolic increase and malignancy, i.e., ^18^F-FDG cannot be considered an infection-specific marker [7,25]. Shavit et al. have used ^18^F-FDG to follow 12 patients with NEO. Eight patients (67%) underwent a second PET/CT scan after active otitis resolved and after at least 6 weeks of antibiotic treatment. The scan demonstrated no or substantially reduced FDG uptake, and treatment was stopped. The patients had no NEO symptoms at the end of the follow-up. One patient had significant uptake, and antibiotic treatment was continued until a third scan demonstrated no FDG uptake. Anatomic methods, such as CT and MRI, may still show signs of positivity if bone erosion and soft tissue involvement persist; 18F-FDG could be useful in determining response to treatment. Despite presenting a higher resolution than the gamma radiation markers, they are also used for conventional hybrid imaging (PET-CT and PET-MRI) for better anatomic visualization [7]. The best practice reported in the literature shows that ^99m^Tc-MDP bone scans and ^67^Ga scintigraphy have been used to diagnose and monitor disease progression in NEO. However, the sensitivity and specificity of these studies are more limited than once imagined. Consequently, ^99m^Tc-MLS, again, must be considered superior for its specificity. If a higher anatomy resolution is needed for disease extension analysis, ^99m^Tc-MLS could be associated with CT or MRI.

## 5. Conclusions

As far as we know, this is the first publication in the world to use this technique in NEO suspicion, which was revealed to be superior in comparison with bone ^99m^Tc-MDP and ^67^Ga scintigraphy regarding exam specificity and is the only one able to provide the needed answers to differ NEO from tumors or chronic inflammation when associated with ^99m^Tc-phytate. Our results suggest that ^99m^Tc-MLS is an excellent diagnostic tool choice that can also be used as therapeutic improvement control as ^67^Ga scintigraphy, which is considered the gold standard.

## Figures and Tables

**Table 1:** Comparative results of the final diagnosis.

Patient	Age	Sex	Side	Bone Scan	Ga-67	Leukocyte Scan	Bone marrow Scan	Diagnosis
1	58	M	R	+	−	+	−	NEO
2	47	M	R	+	+	+	−	NEO
3	56	F	L	+	+	+	−	NEO
4	73	M	L	+	+	+	−	NEO
5	51	M	L	+	+	+	−	NEO
6	21	M	R	+	+	+	+	SARCOMA
7	47	M	R	+	+	+	−	NEO
8	59	F	L	−	−	−	−	NEO
9	14	M	R	+	+	+	−	NEO
10	52	M	R	+	+	+	−	NEO
11	58	F	L	+	−	+	−	NEO
12	68	M	R	+	+	+	−	NEO
13	67	M	R	+	+	+	−	NEO
14	70	M	L	−	+	−	−	−
15	70	M	R	+	+	+	−	NEO
16	42	F	R	+	+	+	−	NEO
17	59	M	L	+	+	+	−	NEO
18	56	F	R	+	+	+	−	NEO
19	38	M	R	+	+	−	+	COLEASTOMA
20	71	M	R	+	+	+	−	NEO
21	49	M	L	+	+	+	−	NEO
22	54	M	R	+	+	+	−	NEO
23	52	M	R	+	+	−	−	−
24	72	M	L	+	+	+	−	NEO

## Data Availability

Data supporting reported results can be found at the archives of the Hospital Universitário Clementino Fraga Filho.
